# Lysosomal Function Is Involved in 17β-Estradiol-Induced Estrogen Receptor α Degradation and Cell Proliferation

**DOI:** 10.1371/journal.pone.0094880

**Published:** 2014-04-15

**Authors:** Pierangela Totta, Valeria Pesiri, Maria Marino, Filippo Acconcia

**Affiliations:** Department of Sciences, Section Biomedical Sciences and Technology, University Roma Tre, Rome, Italy; II Università di Napoli, Italy

## Abstract

The homeostatic control of the cellular proteome steady-state is dependent either on the 26S proteasome activity or on the lysosome function. The sex hormone 17β-estradiol (E2) controls a plethora of biological functions by binding to the estrogen receptor α (ERα), which is both a nuclear ligand-activated transcription factor and also an extrinsic plasma membrane receptor. Regulation of E2-induced physiological functions (e.g., cell proliferation) requires the synergistic activation of both transcription of estrogen responsive element (ERE)-containing genes and rapid extra-nuclear phosphorylation of many different signalling kinases (e.g., ERK/MAPK; PI3K/AKT). Although E2 controls ERα intracellular content and activity via the 26S proteasome-mediated degradation, biochemical and microscopy-based evidence suggests a possible cross-talk among lysosomes and ERα activities. Here, we studied the putative localization of endogenous ERα to lysosomes and the role played by lysosomal function in ERα signalling. By using confocal microscopy and biochemical assays, we report that ERα localizes to lysosomes and to endosomes in an E2-dependent manner. Moreover, the inhibition of lysosomal function obtained by chloroquine demonstrates that, in addition to 26S proteasome-mediated receptor elimination, lysosome-based degradation also contributes to the E2-dependent ERα breakdown. Remarkably, the lysosome function is further involved in those ERα activities required for E2-dependent cell proliferation while it is dispensable for ERα-mediated ERE-containing gene transcription. Our discoveries reveal a novel lysosome-dependent degradation pathway for ERα and show a novel biological mechanism by which E2 regulates ERα cellular content and, as a consequence, cellular functions.

## Introduction

The functions of the cellular proteome are controlled by a homeostatic steady-state, which is granted by the balance between protein synthesis and degradation (*i.e.,* proteostasis). While protein synthesis always requires gene transcription and mRNA translation, cells have evolved different physiological mechanisms to regulate proteolysis and thus protein turnover. Indeed, degradation of intracellular proteins occurs *via* targeted (*i.e.,* ubiquitin-dependent) 26S proteasome activation and extra-cellular proteins are eliminated through a vesicular system that ultimately addresses them to the lysosomes. Remarkably, in recent years, this notion has been refined by the recognition that also intracellular soluble proteins can be shuttled to lysosomes for degradation *via* a non-vescicular system. Thus, beside the homeostatic control of protein turnover, the regulatory mechanisms of proteostasis networks could represent also master organizers of signal transduction circuits [1], [2], [3].

The estrogen receptor α (ERα) is a ligand-activated transcription factor that belongs to the nuclear hormone receptor super-family. ERα, together with the other receptor subtype (ERβ) mediates the pleiotropic effects of the sex hormone 17β-estradiol (E2) that include many physiological processes such as growth, development, and differentiation. In particular, the E2:ERα complex molecular actions are a function of ERα intracellular localization: in the nucleus, the activated ERα drives transcription not only of those genes that contain the estrogen-response element (ERE) within their promoters but also of non-ERE-containing genes through the stimulation of the activity of specific transcription factors (*e.g.,* Sp-1 and AP-1) (*i.e.,* nuclear activity) [4]. Outside of the nucleus, the ERα is extrinsically localized at the plasma membrane. It is now clear that ERα membrane association is required for the E2-dependent activation of rapid kinase signalling pathways (*e.g.,* ERK/MAPK; PI3K/AKT) (*i.e.,* extra-nuclear activity) and the realization of the E2-induced cellular effects both in cell lines (*e.g.,* cell proliferation) [5], [6], [7], [8] and in mice (*e.g.,* cell migration) [9].

The existing paradigm defines that the E2-dependent control of ERα intracellular concentration contributes to the regulation of the pleiotropic effects elicited by E2 in several target tissues. Regulation of ERα stability depends on the activation of the 26S proteasome and is intrinsically connected with the ability of the E2-activated receptor to regulate gene transcription [10], [11], [12]. More recently, we extended this notion by demonstrating that ERα membrane localization and signalling (*e.g.,* PI3K/AKT) also controls E2-induced ERα degradation [6], which can be also activated by exogenous ERα ligands [13].

In addition to 26S proteasome, some relationships among lysosomes and ERα have been reported in different cell lines [14], [15], [16], raising the question of a possible cross-talk among ERα and these intracellular organelles. However, at the present if the localization of endogenous ERα to lysosomes occurs as well as if the lysosomal function could play a role in ERα signalling is still a completely unexplored issue. To this purpose, we studied the role of lysosomes in ERα degradation, E2-dependent signalling and physiological effects in two different breast cancer cell lines (*i.e.,* MCF-7 and T47D-1 mammary adenocarcinoma cells). Our results indicate that ERα degradation requires lysosomal function in addition to the 26S proteasome activity and that lysosomes are implicated in the regulation of the E2-depedent signalling to cell proliferation.

## Results

### The Role of 26S Proteasome in E2-induced ERα Degradation

**Figure 1:**
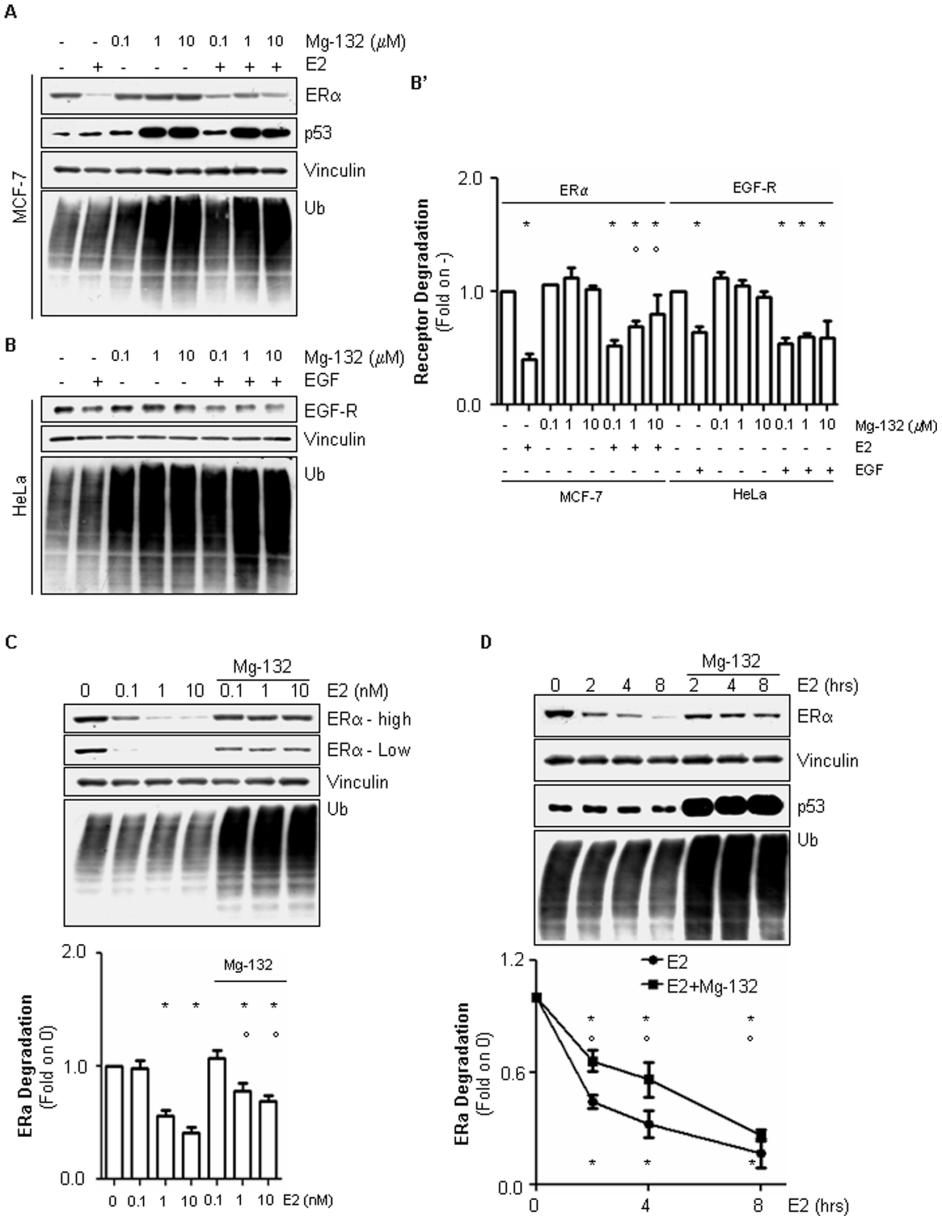
The involvement of 26S proteasome in E2-induced ERα degradation in MCF-7 cells. (A) Western blot of ERα, p53, and ubiquitin (Ub) cellular levels in MCF-7 cells treated for 2 hrs with E2 (10 nM) in the presence of different concentrations of the 26S proteasome inhibitor (Mg-132). (B) Western blot of EGF-R, and ubiquitin (Ub) cellular levels in HeLa cells treated for 2 hrs with EGF (1 µg/ml) in the presence of different concentrations of the 26S proteasome inhibitor (Mg-132). (B’) Densitometric analyses related to ERα and EGF-R protein levels in panel A and B, respectively. (C) Western blot of ERα (high and low exposures) and ubiquitin (Ub) cellular levels in MCF-7 cells treated with the 26S proteasome inhibitor (Mg-132 1 µM) in the presence of different doses of E2. Densitometric analyses of ERα high exposure were performed. (D) Western blot of ERα, p53, and ubiquitin (Ub) cellular levels in MCF-7 cells treated for with E2 (10 nM) at different time points in the presence of the 26S proteasome inhibitor (Mg-132 1 µM). Inhibitor alone was administrated for 2 hours and 30 min. Loading control was done by evaluating vinculin expression in the same filter. *indicates significant differences with respect to the control sample (0 or -); °indicates significant differences with respect to the corresponding E2-treated samples (*p*<0.05). Figure shows representative blots of at least three independent experiments.

In order to understand a potential interplay among lysosomes and ERα, we started by investigating the 26S proteasome-dependent ERα degradation. To this purpose adenocarcinoma (MCF-7) cells were treated for 2 hrs with E2 in the presence or in the absence of the pre-treatment with different doses of Mg-132, an inhibitor of 26S proteasome activity (Fig. 1A). As expected, E2 reduced ERα protein levels in MCF-7 cells and Mg-132 administration prevented in a dose-dependent manner the E2-induced ERα degradation [17]. Surprisingly, the E2-dependent reduction in ERα intracellular levels was only partially reverted by the 26S proteasome inhibitor and the Mg-132 barely affected the basal ERα cellular levels (Fig. 1A and 1B’). Notably, Mg-132 administration (1 and 10 µM) to MCF-7 cells effectively induced the accumulation of total cellular ubiquitinated species as well as the increase in the rapidly 26S proteasome turn-overed protein p53 (Fig. 1A and 1B’), thus demonstrating the 26S proteasome is actually inhibited by the drug treatment. Remarkably, 10 µM Mg-132 is toxic and induces cell death in MCF-7 cells (data not shown). Moreover, epidermal growth factor (EGF)-induced EGF receptor (EGF-R) degradation, which occurs in the lysosomes [18], was not affected by Mg-132 doses that were effective in increasing the total cellular ubiquitinated species in HeLa cells (Fig. 1B and 1B’). Prompted by these observations, we next performed experiments to evaluate the dose- and time-dependent effect of E2 on ERα breakdown in MCF-7 cells in the presence of 1 µM of Mg-132 (Fig. 1A). In line with the previous results, treatment of MCF-7 cells with Mg-132 prevented but did not completely block the dose- (Fig. 1C – high exposure and low exposure) and time-induced (Fig. 1D) E2-dependent reduction in ERα intracellular levels. Notably, 1 µM Mg-132 administration efficiently induced the accumulation both of total cellular ubiquitinated species (Fig. 1C and 1D) and of the 26S proteasome-degraded protein p53 also at longer time points (Fig. 1D).

These data confirm that ERα breakdown is regulated by the 26S proteasome but additionally suggest the presence of other degradation mechanisms in the control of ERα intracellular levels.

### The Role of Lysosomes in E2-induced ERα Degradation

Because we observed that the 26S proteasome activity is only partially required for E2-induced ERα degradation, we next studied the role of lysosomes in the control of ERα intracellular level. To fulfil this task, we evaluated if ERα could localize to lysosomes by employing an ERα antibody, which highlights cytoplasmic ERα in breast cancer cells (*i.e.,* Sp-1 ERα) [19].

**Figure 2:**
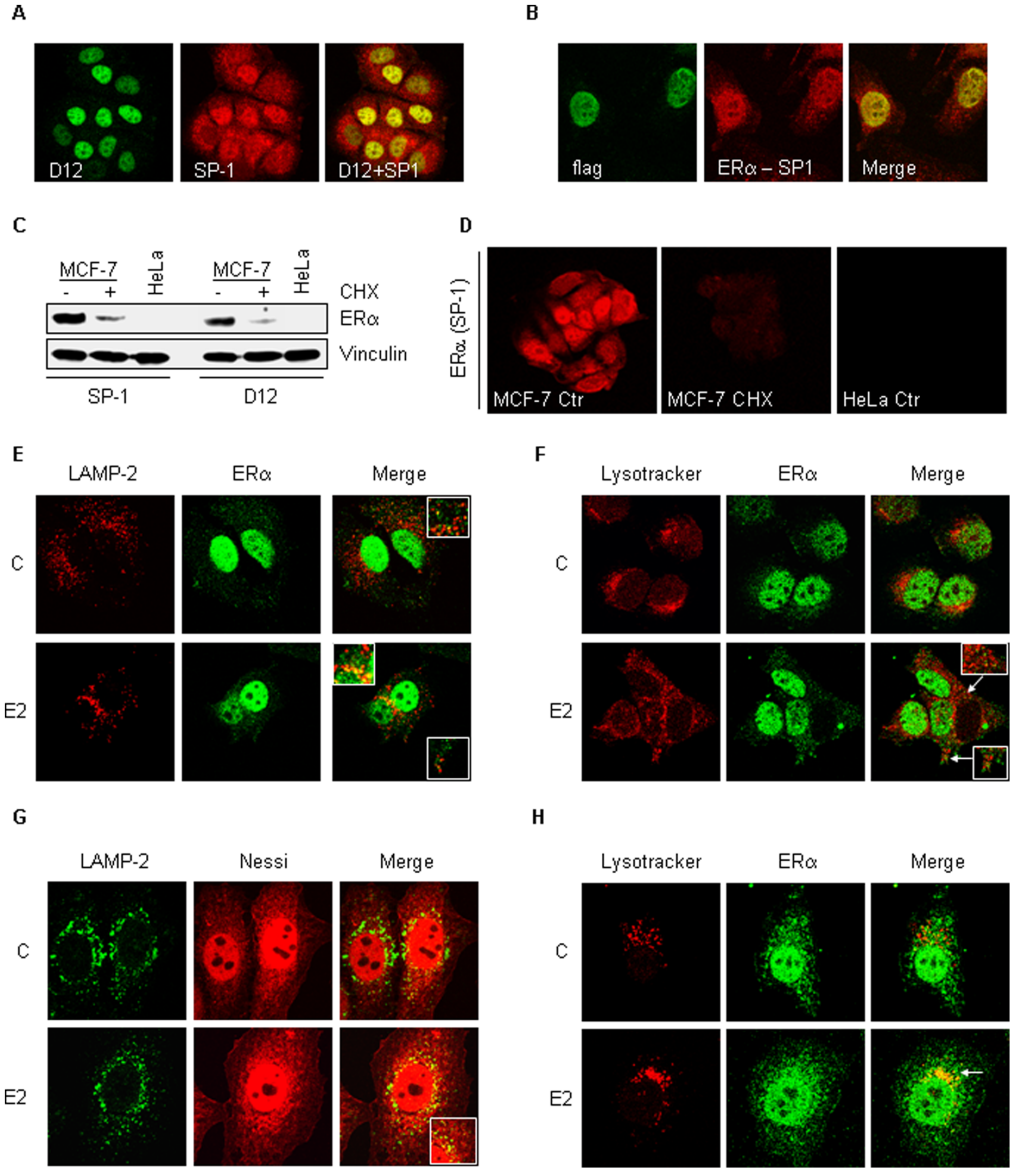
ERα localization to lysosomes. (A) Growing MCF-7 cells were co-stained with anti-ERα Sp-1 and D-12 antibodies. (B) Growing pc DNA flag ERα -transfected HeLa cells were co-stained with anti-ERα HC-20 and flag antibodies. (C) Western blot analysis of ERα cellular levels in growing MCF-7 cells treated with cycloheximide (CHX - 1 mg/ml) for 24 hrs with both anti-ERα Sp-1 and D-12 antibodies. Loading control was done by evaluating vinculin expression in the same filter. (D) Anti-ERα Sp-1 staining of MCF-7 cells treated for with cycloheximide (CHX - 1 mg/ml) for 24 hrs. MCF-7 cells were co-stained with anti-ERα Sp-1 antibody together with either LAMP-2 antibody (E) or lysotracker (F) both in the presence and in the absence of E2 (10 nM–2 hrs). pc DNA flag ERα (Nessi)-transfected HeLa cells were co-stained with anti-ERα HC-20 antibody together with either LAMP-2 antibody (G) or lysotracker (H) both in the presence and in the absence of E2 (10 nM–2 hrs). Figures show one unique confocal plane. All co-staining procedures were described in details in the Material and Methods section.

Initial experiments were performed to test the specificity of the anti-ERα Sp-1 antibody, which recognizes an epitope located within the ERα *C*-terminus, in comparison with another anti-ERα antibody (*i.e.,* D12), which recognizes an epitope located within the ERα *N*-terminus. Confocal microscopy analysis demonstrated that the anti-ERα Sp-1 antibody stains MCF-7 cells both in the nucleus and in the cytoplasm while anti-ERα D12 antibody stains only the nucleus of MCF-7 cells (Fig. 2A, left and middle panel). Remarkably, treatment of MCF-7 cells with both anti-ERα antibodies showed a perfect nuclear co-staining (Fig. 2A, right panel). Accordingly, treatment of transfected flag-tagged ERα HeLa cells with both anti-flag and anti-ERα Sp-1 antibodies demonstrated only co-staining of cell nuclei (Fig. 2B). Thus, the anti-ERα Sp-1 antibody recognizes both the endogenous and the over-expressed ERα. To further confirm that the cytoplasmic staining observed in cells treated with anti-ERα Sp-1 antibody is indeed the ERα located outside of the nucleus, Western blot analysis and immunoflorescence staining were performed in MCF-7 cells treated for 24 hrs with the protein translation inhibitor cycloheximide (CHX) in order to reduce ERα cellular content [13]. In MCF-7 cells, anti-ERα Sp-1 and D12 antibodies were equally able to detect the CHX-dependent reduction in ERα cellular levels by Western blotting (Fig. 2C) and the anti-ERα Sp-1 antibody-dependent staining was decreased both in the nucleus and in the cytoplasm of CHX-treated MCF-7 cells (Fig. 2D). All anti-ERα antibodies failed to detect any signal in ERα-negative HeLa cells (Fig. 2C and 2D). These results confirm that the anti-ERα Sp-1 antibody is specific for the ERα and clearly recognizes the same nuclear ERα in the cytoplasm of breast cancer cells [19]. Therefore, as previously reported [20], antibodies against different *N*- and *C*-terminus ERα epitopes stain ERα either only in the nucleus or in the nucleus and in the cytoplasm, respectively.

Anti-ERα Sp-1 antibody was used to stain MCF-7 cells together with markers of lysosomes (*i.e.,* LAMP-2 and lysotracker). ERα barely co-localizes with LAMP-2 and lysotraker in MCF-7 cells under basal conditions while E2 treatment determines a time-dependent increase in cytoplasmic co-localization of ERα with either lysosomal markers that reached a maximum after 2 hrs of hormone treatment (Fig. 2E, 2F and data not shown). Accordingly, the same results were obtained in another ERα-positive breast cancer cell line (T47D-1) (Fig. S1A and S1B). Parallel experiments were also conducted in HeLa cells transiently transfected with an ERα mutant (*i.e.,* H2_NES - Nessi) with increased ERα cytoplasmic localization (Fig. S2A and S2B) [21]. Also in ERα Nessi-transfected HeLa cells, 2 hrs of E2 treatment was able to increase the cytoplasmic co-localization of ERα with LAMP-2 and lysotracker (Fig. 2G and 2H).

**Figure 3:**
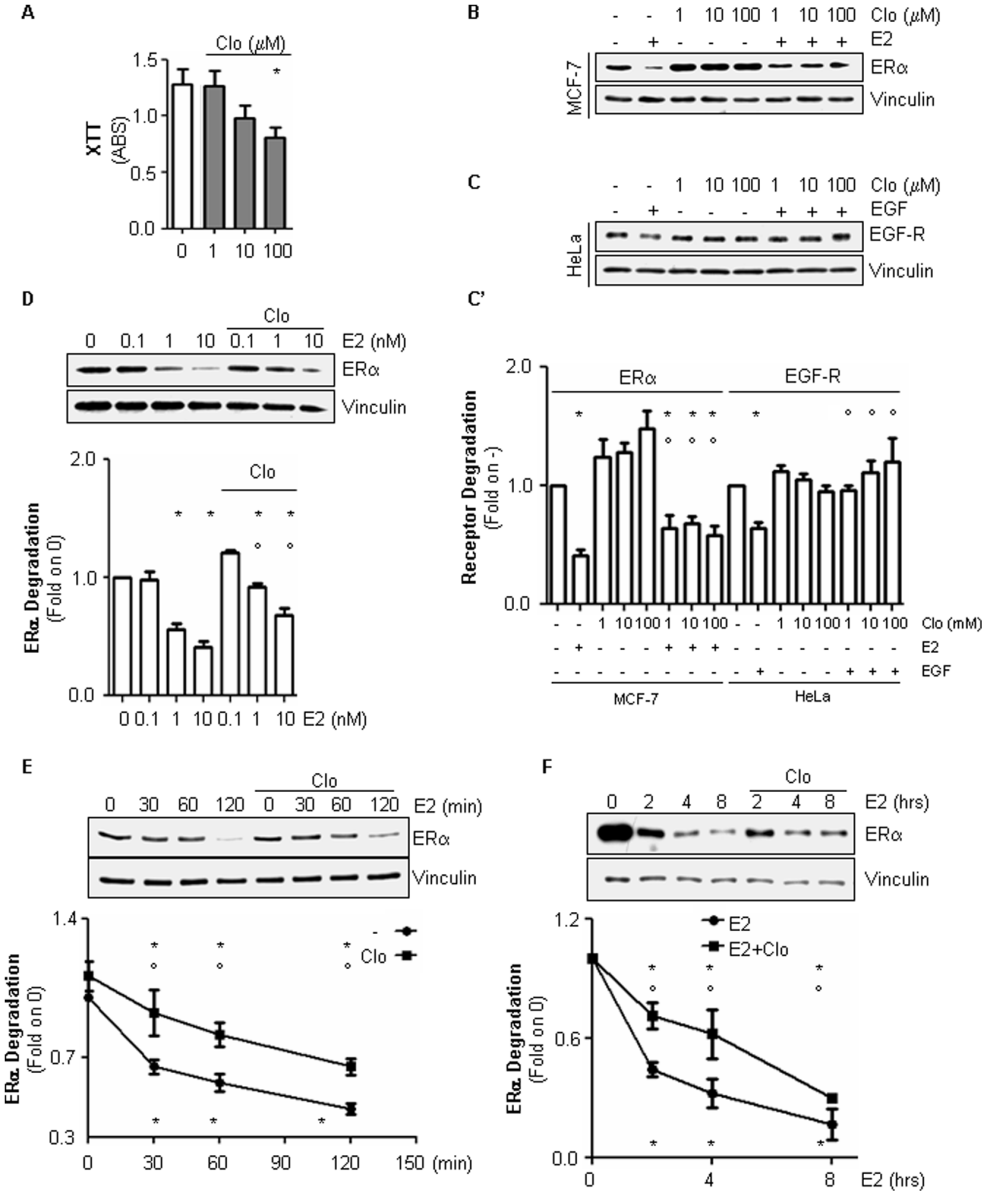
The involvement of lysosomes in E2-induced ERα degradation in MCF-7 cells. (A) XTT assay in growing MCF-7 cells treated for 24 hrs with different doses of chloroquine (Clo). (B) Western blot and relative densitometric analyses (C’) of ERα cellular levels in MCF-7 cells treated for 2 hrs with E2 (10 nM) in the presence of different concentrations of chloroquine (Clo). (C) Western blot and relative densitometric analyses (C’) of EGF-R cellular levels in HeLa cells treated for 2 hrs with EGF (1 µg/ml) in the presence of different concentrations of chloroquine (Clo). (D) Western blot and relative densitometric analyses of ERα cellular levels in MCF-7 cells treated with chloroquine (Clo 10 µM) in the presence of different doses of E2. (E–F) Western blot and relative densitometric analyses of ERα cellular levels in MCF-7 cells treated for with E2 (10 nM) at different time points in the presence of chloroquine (Clo 10 µM). Inhibitor alone was administrated for 2 hours and 30 min. Loading control was done by evaluating vinculin expression in the same filter. *indicates significant differences with respect to the control sample (0 or -); °indicates significant differences with respect to the corresponding E2-treated or EGF-treated samples (*p*<0.05). Figure shows representative blots of at least three independent experiments.

In order to study the involvement of lysosomes in ERα degradation, chloroquine (Clo), a drug that inhibits lysosomal enzymes by changing endosomes and lysosomes internal pH [22], has been used. Two hrs after E2 treatment ERα protein levels were reduced by 60% both in a time- (Fig. 3B, 3E and 3F) and dose- (Fig. 3D) dependent manner in MCF-7 cells. Pre-treatment of MCF-7 cells with different doses of Clo revealed that this drug is able to partially block the E2-dependent reduction in ERα intracellular levels (Fig. 3B and 3C’), thus indicating that ERα degradation requires at least in part an intact lysosomal function. As expected [18], EGF-induced EGF-R degradation was prevented by Clo in a dose-dependent manner in HeLa cells (Fig. 3C and 3C’). In addition, treatment of MCF-7 cells with doses of Clo (*i.e.,* 10 µM) that barely affect MCF-7 cell viability (Fig. 3A) induced a dose- (Fig. 3D) and time- (Fig. 3E and 3F) dependent blockade of the E2-evoked reduction in ERα cellular content. Note that, Clo administration did not significantly modify the basal cellular content of ERα and EGF-R in MCF-7 and HeLa cells, respectively (Fig. 3B, 3C, 3C’ and 3E). Remarkably, similar results were obtained in T47D-1 cells (Fig. S1C).

**Figure 4:**
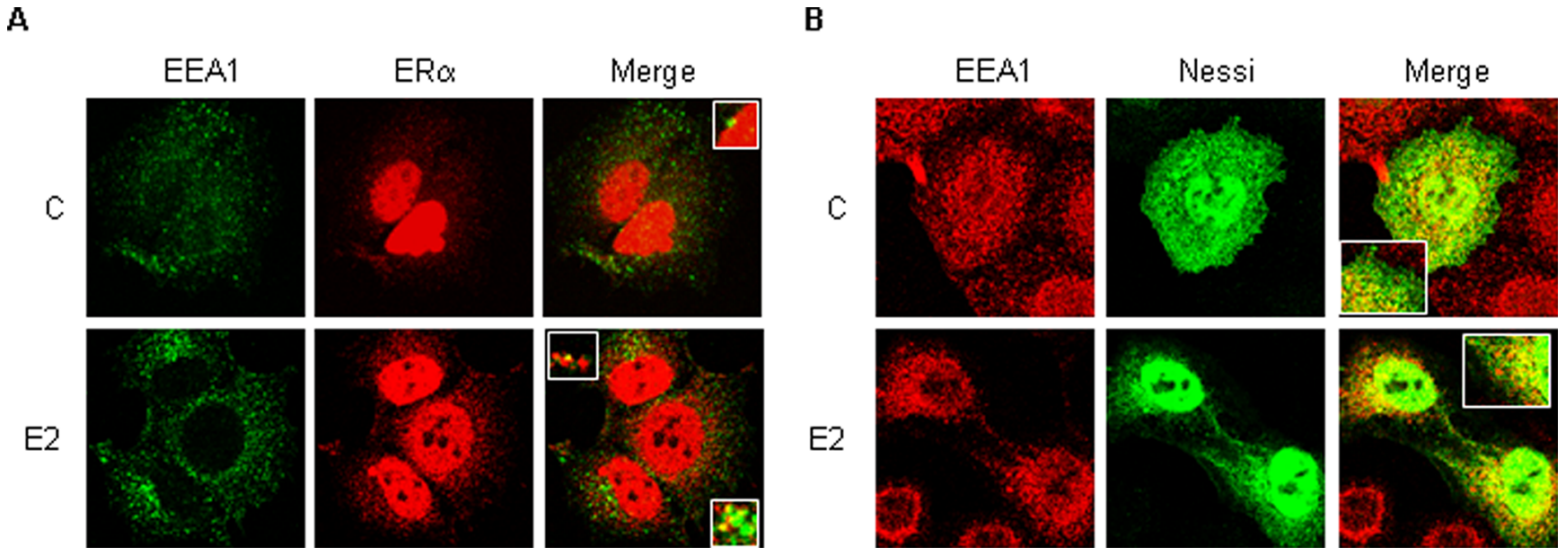
ERα localization to early endosomes. (A) MCF-7 cells were co-stained with anti-ERα Sp-1 and EEA1 N-19 antibodies both in the presence and in the absence of E2 (10 nM–15 min). pc DNA flag ERα (Nessi)-transfected HeLa cells were co-stained with anti-ERα HC-20 and EEA1 H-300 antibodies both in the presence and in the absence of E2 (10 nM–15 min). Figures show one unique confocal plane. All co-staining procedures were described in details in the Material and Methods section.

The change in endosomal pH caused by chloroquine has also the consequence to impede the fusion of endosomes to lysosomes [22]. Because ERα localizes to lysosomes (Fig. 2), we reasoned that ERα could be addressed at the endosomal compartment. Confocal microscopy analysis demonstrated that upon 15 min of E2 administration ERα co-localized with the early endosomal antigen (EEA1) both in MCF-7 cells (Fig. 4A) and in Nessi-transfected HeLa cells (Fig. 4B). Interestingly, only minor endosomal localization was observed in un-treated cells (Fig. 4) and after 2 hrs of E2 treatment (data not shown).

Altogether, these data strongly indicate that cytoplasmic ERα is addressed to the lysosomal compartment in an E2-dependent manner and that lysosomal function is implicated in the control of ERα cellular content.

### The Role of Lysosomes in Nuclear and Extra-nuclear ERα-dependent Signalling

**Figure 5:**
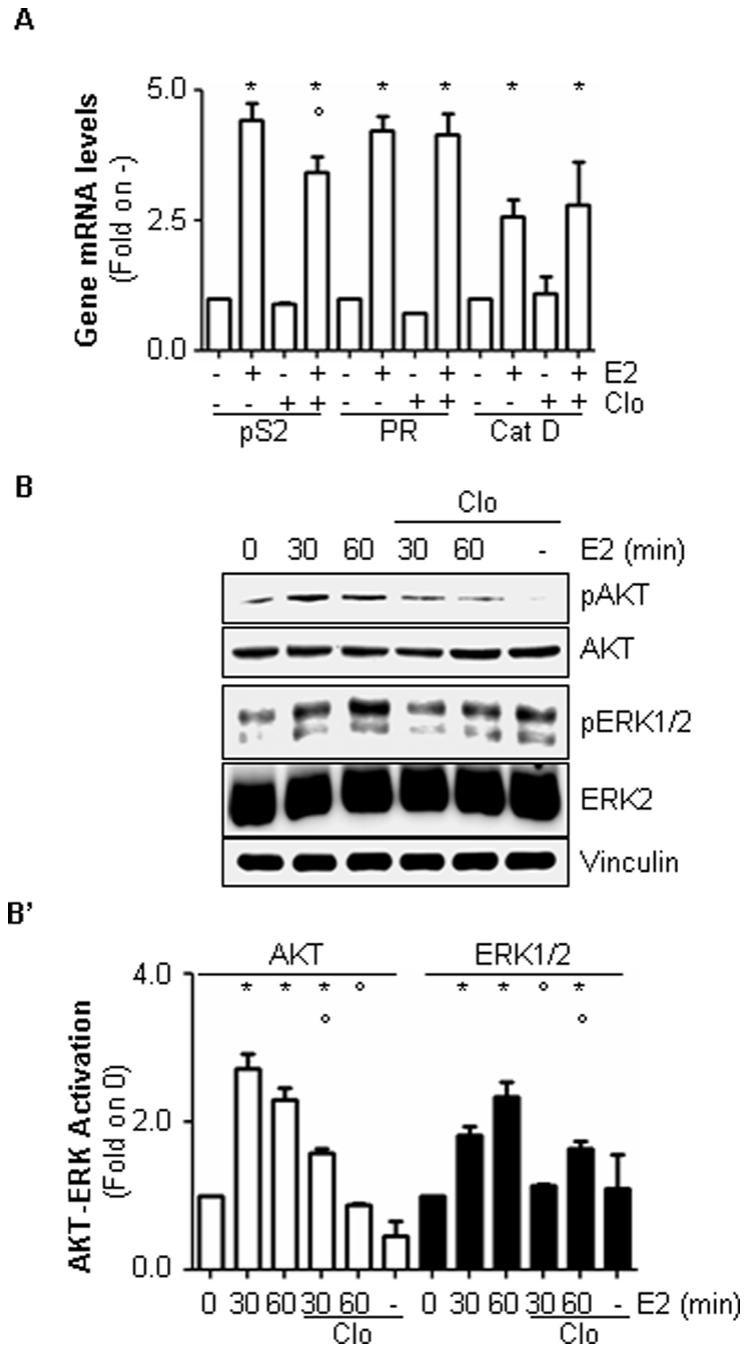
The involvement of lysosomes in E2-induced ERα activities. (A) RT-qPCR analysis of pS2/TIFF (pS2), cathepsin D (CatD) and progesterone receptor (PR) mRNA expression normalized on the GAPDH mRNA expression in MCF-7 cells treated with E2 (10 nM) for 24 hrs both in the presence and in the absence of chloroquine (Clo–10 µM) treatment. Western blot (B) and relative densitometric (B’) ERK1/2 and AKT phosphorylation in MCF-7 cells treated with E2 (10 nM) at different time points. Where indicated, cells were treated chloroquine (Clo–10 µM) 30 min before E2 administration. Loading control was done by evaluating vinculin expression in the same filter. *indicates significant differences with respect to the relative control (0) sample; °indicates significant differences with respect to the corresponding E2 sample (*p*<0.05). Figure shows representative blots of three independent experiments.

Because ERα degradation contributes to E2-induced ERα gene transcription [11], [12], we next studied the impact of lysosomal function on E2-dependent ERα transcriptional activity. Real-time qPCR analysis revealed that in MCF-7 cells pre-treatment with Clo does not prevent the increase in the amount of the E2-responsive ERE-containing gene cathepsin D (Cat D) and progesterone receptor (PR) mRNA levels observed after 24 hrs of E2 administration (Fig. 5A). On the other hand, Clo treatment slightly but significantly reduced the E2-induced presenelin 2 (pS2) mRNA accumulation in MCF-7 cells (Fig. 5A). These data demonstrate that lysosomal function is dispensable for nuclear ERα ERE-based gene transcription.

It is now accepted that the extra-nuclear plasma membrane localized ERα directs the activation of the rapid E2 signalling *in vitro* and *in vivo* [5], [6], [8], [9], [23]. In particular, although many different signal transduction pathways are rapidly activated upon E2 administration, the ERK/MAPK and PI3K/AKT pathways seem to be the main extra-nuclear induced signalling cascades in breast cancer cells [4]. Indeed, time-course analysis revealed that E2 induces a rapid increase in ERK1/2 and AKT phosphorylation in MCF-7 cells that was strongly reduced by 10 µM Clo pre-treatment. Notably, no significant changes in the basal ERK1/2 and AKT phosphorylation and total cellular levels were detected under Clo administration (Fig. 5B and 5B’). These data demonstrate that lysosomal function modulates E2-induced ERα-mediated ERK1/2 and AKT extra-nuclear activation.

### The Role of Lysosomes in E2-induced Breast Cancer Cell Proliferation

**Figure 6:**
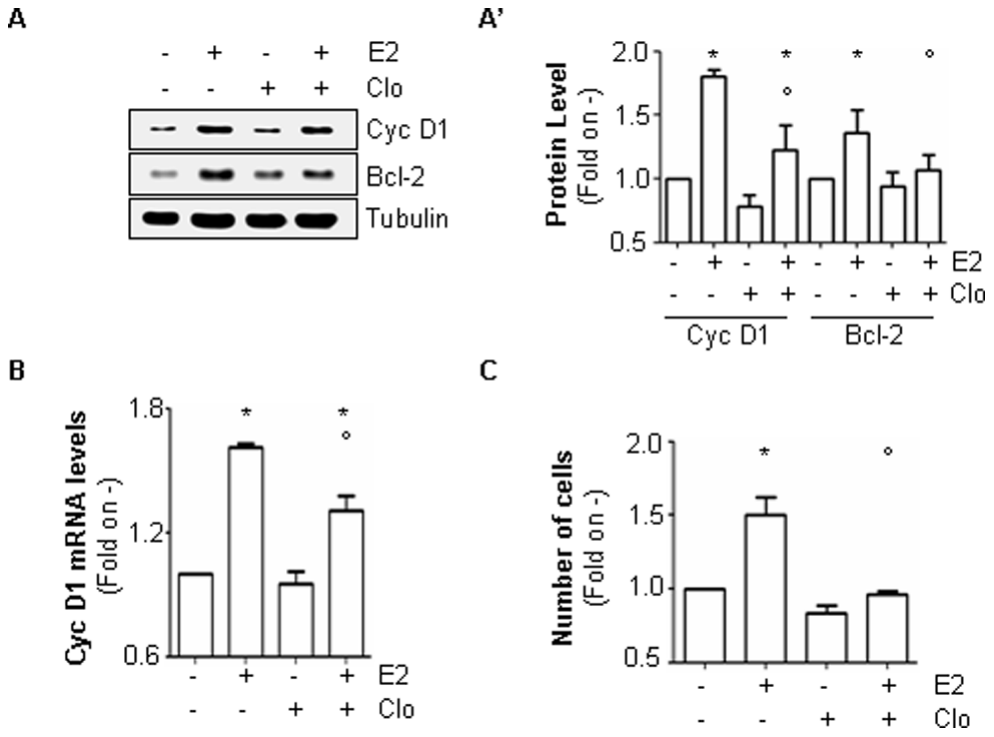
The involvement of lysosomes in E2-induced cell proliferation. Western blot (A) and relative densitometric (A’) analyses of cyclin D1 (Cyc D1) and Bcl-2 expression levels in MCF-7 cells treated with E2 (10 nM–24 hours) both in the presence and in the absence of chloroquine (Clo–10 µM). Loading control was done by evaluating tubulin expression in the same filter. *indicates significant differences with respect to the relative control (−) sample; ° indicates significant differences with respect to the corresponding E2 sample (*p*<0.05). Figure shows representative blots of three independent experiments. (B) RT qPCR analysis of cyclin D1 (Cyc D1) mRNA expression normalized on the GAPDH mRNA expression in MCF-7 cells treated with E2 (10 nM–24 hours) both in the presence and in the absence of chloroquine (Clo–10 µM). (C) Number of MCF-7 cells treated with E2 (10 nM–48 hours) both in the presence and in the absence of chloroquine (Clo–10 µM). *indicates significant differences with respect to control (−); °indicates significant differences with respect to E2 sample (*p*<0.01).

Many groups including our own have clarified that the E2-induced ERα extra-nuclear activity is required for E2-induced cell proliferation. In particular, the E2-dependent activation of ERK/MAPK and PI3K/AKT pathways control the transcription of specific cell cycle regulated genes (*e.g.,* cyclin D1) and in parallel up-regulate the level of the anti-apoptotic and pro-survival protein Bcl-2 [5], [8], [24], [25]. Thus, cyclin D1 and Bcl-2 expression was evaluated in MCF-7 cells both in the presence and in the absence of Clo. Interestingly, the E2-dependent induction in cyclin D1 mRNA and protein levels as well as in Bcl-2 cellular content was significantly reduced by Clo in MCF-7 cells (Fig. 6A, 6A’ and 6B) as well as in T47D-1 cells (Fig. S1D). In line with these results, E2 treatment was able to induce a significant increase in the cell number with respect to un-stimulated MCF-7 (Fig. 6C) and T47D-1 (Fig. S1E) cells. On the contrary, E2 did not trigger cell proliferation in either breast cancer cell lines when the hormone was administrated in the presence of Clo (Fig. 6C and Fig. S1E). Therefore, lysosomal function plays a critical role in the regulation of E2:ERα-mediated cell proliferation.

## Discussion

The mechanisms that govern the homeostasis of the cellular proteome are fundamental for the regulation of cellular processes. Consequently, protein cellular content is strictly controlled by protein synthesis and degradation systems in order to guarantee the selected protein function [1], [2], [3]. Regulation of protein abundance is particularly critical for receptors for hormones and growth factors. Indeed, the hormone binding to its receptor often results in receptor down-regulation, which occurs in parallel with the hormone-dependent regulation of the functional effects and is required for de-sensitization of target cells to the hormone. Depending on the nature of the receptor, down-regulation-dependent receptor degradation can take place either through the 26S proteasome or in the lysosomes [1].

Also in ERα-containing cells the exposure to 17β-estradiol (E2) results in a ligand-dependent reduction of the total receptor content. In this way, E2 determines the amount of ERα intracellular levels by controlling receptor turnover and synchronizes ERα activities with the cellular response. The mechanism underlying ERα elimination requires the activation of the 26S proteasome. Indeed, both apoERα and E2-activated receptor undergo proteasomal degradation [26]. The data presented here confirm that ERα degradation is under the control of 26S proteasome activity. In addition, we found that the inhibition of 26S proteasome does not completely prevent ERα elimination (Fig. 1). These results are in contrast with those demonstrating how the 26S proteasome inhibition blocks E2-induced ERα breakdown {for reviews please see [10], [26]}. However, this notion relies on experiments performed by administrating cells with very high concentrations of Mg-132 (ranging from 10 to 50 µM) {see for example [12], [17], [27]}. In our hand, these doses of Mg-132 are toxic and induce cell death in MCF-7 cells. On the contrary, lower doses of the 26S proteasome inhibitor (*i.e.,* 1 µM) do not affect breast cancer cell viability and are the minimum sufficient amount that determines the time- and dose-dependent (Fig. 1) accumulation of total polyubiquitinated species and the increase in the cellular content of p53, another transcription factor that rapidly undergoes proteasomal degradation [18]. Moreover, 1 µM Mg-132 is ineffective in inhibiting the degradation of EGF-R (Fig. 1B), a membrane receptor that is degraded in lysosomes [18]. Thus, in our experimental settings, under conditions in which the 26S proteasome is efficiently inhibited, the E2-induced ERα breakdown is only partially prevented (Fig. 1). In turn, we conclude that 26S proteasomal degradation regulates ERα intracellular levels but other pathways could affect E2-induced ERα degradation.

Accordingly, we report here that lysosomes contribute to the E2-dependent control of ERα intracellular content (Fig. 3). Indeed, administration of non-toxic doses of chloroquine (*i.e.,* 10 µM) (Fig. 3A), which are effective in blocking the lysosomal-dependent EGF-induced EGF-R degradation {(Fig. 3C) and [28]}, partially prevents the dose- (Fig. 3D) and time-dependent (Fig. 3E, 3F and Fig. S1C) E2-induced ERα degradation in breast cancer cells. Several lines of evidence have suggested a putative interplay among lysosomes and ERα signalling. Indeed, biochemical experiments performed in rat uterine cells showed that radioactive E2 has been found in sub-cellular fractions corresponding to the lysosome-enriched compartment [14], gold-labelled E2 conjugated with BSA (*i.e.,* gold E2-BSA) was observed in lysososmes of HepG2 cells by electron microscopy [15] and fluorescent labelled E2-BSA or transfected GFP-tagged ERα were separately shown to co-localize with lysotracker in NR-38 neurons [16]. Moreover, the glucocorticoid receptor degradation was found to be partially dependent on lysosomes in modified human embryonic kidney AD293 cells [29]. Thus, lysosome-based degradation contributes to the regulation of the cellular content of both ERα and other nuclear receptors.

The accumulation of ERα observed in the presence of the lysosome-distrupting function drug chloroquine rapidly occurs after E2 administration (30 min) and remains significant up to 8 hrs of hormone treatment (Fig. 3E and 3F), possibly implicating lysosomes also in nuclear and extra-nuclear ERα activities. Interestingly, data obtained in MCF-7 cells demonstrate that the lysosome function is dispensable for ERE-containing gene transcription (Fig. 5A) while it is necessary for the activation of the E2-induced ERα-mediated extra-nuclear effects (Fig. 5B). At the present, the mechanistic reasons underlying this different role of lysosomes in ERα activities are not clear. However, it is possible that E2-induced lysosomal-dependent ERα degradation is not required for the nuclear ERα promoter shuttling and transcriptional activity for which 26S proteasome is instead necessary [11], [12], [30] while cytoplasmic ERα lysosomal degradation could sustain signalling. Lysosomal function, which serves the compartmentalized degradation of protein [31], is necessary for E2-induced ERK/MAPK and PI3K/AKT pathway activation and the interference with lysosome integrity severely impairs the E2-dependent proliferation effect in MCF-7 and T47D-1 cells. These findings confirm that the activation of these signalling cascades requires at least in part an intact lysosomal function [31] and further sustain the interdependency between ERα extra-nuclear signalling and E2 cell proliferation [4]. Moreover, our data indicate that lysosomes play a critical role in the E2-induced extra-nuclear events, which drive breast cancer cells to proliferate. Present results demonstrate that chloroquine limits the ability of E2 to trigger both the up-regulation of the cell cycle regulating gene cyclin D1 and of the anti-apoptotic and pro-survival protein Bcl-2 (Fig. 6). In turn, chloroquine treatment impedes E2 to induce breast cancer cell proliferation (Fig. 6 and Fig. S1D and S1E). Interestingly, chloroquine is the election drug approved for treatment of humans affected by malarial disease [31]. Therefore, in light of re-purposing old drugs for novel pharmacological targets [32], this anti-malarial molecule could be in principle used for treatment of ERα-positive breast cancers [31]. However, these findings indicate that lysosomal function takes part in ERα signalling pathways that mediate physiological hormone-induced effects.

Another finding presented here is the fact that the cytoplasmic ERα localizes at the lysosomes (Fig. 2 and Fig. S1A and S1B). This statement is supported by the observations obtained by employing either ERα antibody that specifically recognizes ERα outside of the nucleus of breast cancer cells (*i.e.,* anti-ERα Sp-1 antibody) {[19] and present results} or an ERα mutant (*i.e.,* H2_NES - Nessi) that is abundantly and artificially located in the cytoplasm {[21] and present results}. The use of these reagents allowed us to overcome the caveat that most of the available antibodies cannot detect cytoplasmic ERα [19] and to confirm that ERα assumes different conformations in different intracellular compartments {[20] and present results}. Notably, this evidence further corroborates the notion that the extra-nuclear localized ERα is the same protein as the nuclear-localized receptor [5], [6], [8], [9]. Furthermore, we report that the activated ERα localizes to early endosomes (Fig. 4). E2 rapidly (15 min) determines the localization of the cytoplasmic ERα to the early endosomes (Fig. 4) and prolonged E2 treatment (*i.e.,* up to 2 hrs) also induces a progressive reduction in the co-localization of the ERα with the EEA1 (Fig. 4). In parallel, a progressive co-localization of the receptor with LAMP-2, which peaks after 2 hrs of hormone treatment (Fig. 2), occurs. Because early endosomes are sorting vesicles where cargoes are subjected to distinct trafficking paths that ultimately determine if they will undergo degradation in the lysosome or recycle to the plasma membrane [18], our data strongly suggest that E2-activated cytoplasmic ERα is rapidly routed to endosomes and than to lysosomes. Although it is well established that only extra-cellular and trans-membrane proteins are targeted for degradation to the lysosomes through the activation of specific endocytic routes [18], in recent years it has become clear that also cytoplasmic proteins can be addressed to the lysosomes for degradation [3]. In particular, LAMP-2, which is located at the membrane of lysosomes, works as a molecular pump that allows the up-take of cytoplasmic proteins into the lysosomal lumen [3]. Thus, E2-dependent ERα co-localization with LAMP-2 (Fig. 2) further suggests that the receptor is up-taken into lysosomes for degradation. Nonetheless, the possibility that at the endosomes the E2:ERα complex could be sorted for recycling or to other fates cannot be excluded. Regarding the mechanism that addresses the E2-activated ERα to the endosomoes, it has to be mentioned that all endocytic internalization pathways (*e.g.,* clathrin-mediated and caveolin-mediated endocytosis) that originate from the plasma membrane feed in the endosomes [18]. Thus, it is tempting to speculate an endocytosis-dependent ERα trafficking in breast cancer cells for which membrane E2-loaded ERα [6], [8], [9] could be internalized through different endocytic routes [18] and shuttled to specific intracellular compartment (*e.g.,* lysosomes; nucleus). In this respect, published evidence suggests both an active mechanism for E2 internalization into cells and an endocytic shuttling for the membrane-localized ERα [2], [5], [6], [14], [33], [34], [35]. Nonetheless, this hypothesis, which is currently being tested in our lab, remains to be demonstrated.

In conclusion, the findings reported here reveal a novel role for lysosomes in E2-induced ERα degradation as well as in those ERα activities required for E2-dependent breast cancer cell proliferation. Remarkably, our data, together with the recognition that the activity and the cellular concentration of the receptor for glucocorticoids are at least in part under the control of lysosomes [29], show a new mechanism by which E2 regulates ERα cellular content and further support a novel model of nuclear receptor degradation.

## Materials and Methods

### Cell Culture and Reagents

Human breast adenocarcinoma cells (MCF-7 and T47D-1) [6]. 17β-estradiol, epidermal growth factor (EGF), DMEM (with and without phenol red) and charcoal-stripped fetal calf serum were purchased from Sigma-Aldrich (St. Louis, MO). Bradford protein assay was obtained from Bio-Rad (Hercules, CA). Antibodies against ERα (HC-20 rabbit; D-12 mouse), ubiquitin (P4D1 mouse), p53 (DO-1 mouse), cyclin D1 (H-295 rabbit), phospho-ERK1/2 (E4 mouse), ERK2 (C14 rabbit), Bcl-2 (C2 mouse), LAMP-2 (H4B4 mouse) and EEA1 (N-19 goat) or EEA1 (H-300 rabbit) were obtained from Santa Cruz Biotechnology (Santa Cruz, CA); anti-vinculin and anti-tubulin antibodies were purchased from Sigma-Aldrich (St. Louis, MO). Anti-phospho-AKT and anti-AKT antibodies were purchased from Cell Signaling Technology Inc. (Beverly, MA). Anti-ERα Sp-1 antibody was purchased from Thermoscientific (Waltham, MA, USA). The anti-EGF receptor antibody (rabbit) was a generous gift of Dr Sara Sigismund – IFOM – The FIRC Institute for Molecular Oncology [28]. Chemiluminescence reagent for Western blot was obtained from Biorad Laboratories (Hercules, CA, USA). XTT assay kit was purchased by Roche (Indianapolis, IN, USA) and used according to manifacturer’s instructions. The 26S proteasome inhibitor, Mg-132, was purchased by Calbiochem (San Diego, CA). All the other products were from Sigma-Aldrich. Analytical- or reagent-grade products, without further purification, were used.

### Cellular and Biochemical Assays

Cells were grown in 1% charcoal-stripped fetal calf serum medium for 24 h and then stimulated with E2 at the indicated time points; where indicated, inhibitors (Mg-132; chloroquine) were added 30 min before E2 administration. Unless otherwise indicated, cell were treated with E2 (10^−8^ M), Mg-132 (1 µM), chloroquine (Clo) (10 µM) or EGF (1 µg/ml). Cell number counts, protein extraction, biochemical assays were performed as previously described [6]. Western blot analysis were performed as in [6] but for the transfer procedure: proteins were transferred onto pre-casted nitrocellulose or PVDF membranes using the trans-blot turbo transfer system (Biorad Laboratories, Hercules, CA, USA) for 10 min at room temperature. Band acquisition was performed by using the C-Digit Blot Scanner (Li-Cor Lincon, NE, USA).

### Plasmids and Transient Transfection

The pcDNA 3.1 flag-ERα was previously described [6]. The pcDNA flag-ERα H2_NES mutant (Nessi) was synthesized by GenScript USA Inc. by introducing the R256A,K257A,R259A,R260A,R263A,K266A,K268A,R269L,R271A,D272L mutations within the ERα hinge region in the BamHI/XhoI pcDNA 3.1 flag C sites and sequence verified. Details are available upon request. This receptor variant has all the nuclear localization signals mutated and a nuclear export signal has been introduced in order to increase ERα cytoplasmic localization [21]. HeLa cells were grown to 70% confluence and then transfected using lipofectamine reagent according to the manufacturer’s instructions [6].

### RNA Isolation and qPCR Analysis

The sequences for gene-specific forward and reverse primers were designed using the OligoPerfect Designer software program (Invitrogen, Carlsbad, CA, USA). The following primers were used: for human pS2 5′-CATCGACGTCCCTCCAGAAGAG-3′ (forward) and 5′-CTCTGGGACTAATCACCGTGCTG-3′ (reverse), for human cyclin D1 5′- AACTACCTGGACCGCTTCCT-3′ (forward) and 5′- CCACTTGAGCTTGTTCACCA-3′ (reverse), for human cathepsin D 5′-GTACATGATCCCCTGTGAGAAGGT-3′ (forward) and 5′-GGGACAGCTTGTAGCCTTTGC-3′ (reverse), for human progesterone receptor (PR) 5′-AAATCATTGCCAGGTTTTCG-3′ (forward) and 5′-TGCCACATGGTAAGGCATAA-3′ (reverse), for human GAPDH 5′-CGAGATCCCTCCAAAATCAA-3′ (forward) and 5′-TGTGGTCATGAGTCCTTCCA-3′ (reverse). Total RNA was extracted from cells using TRIzol Reagent (Invitrogen, Carlsbad, CA, USA) according to the manufacturer’s instructions. To determine gene expression levels, cDNA synthesis and qPCR were performed using the GoTaq 2-step RT-qPCR system (Promega, Madison, MA, USA) in a ABI Prism 7900 HT Sequence Detection System (Applied Biosystems, Foster City, CA, USA) according to the manufacturer’s instructions. Each sample was tested in triplicate, the experiment repeated twice and the gene expression normalized for GAPDH mRNA levels.

### Confocal Microscopy Analysis

MCF-7, T47D-1 and ERα-transfected HeLa cells were plated and stained as previously described [36]. Briefly, cells were grown on 30-mm glass cover slips and than fixed with paraformaldehyde (4%). For anti-ERα Sp-1 (1∶1000) and D-12 (1∶30) co-staining (Fig. 2A) cells were permeabilized with Triton-X 100 (0.1%) in PBS for 1 min. For anti-ERα HC-20 (1∶30) and flag (1∶10000) co-staining (Fig. 2B and Fig. S2B) cells were permeabilized with Triton-X 100 (0.1%) in PBS for 5 min. For anti-ERα Sp-1 (1∶1000) and LAMP-2 (1∶100) co-staining (Fig. 2E and Fig. S1A) cells were permeabilized with Triton-X 100 (0.1%) in PBS for 1 min. For anti-ERα HC-20 (1∶30) and LAMP-2 (1∶100) co-staining (Fig. 2G) cells were permeabilized with Triton-X 100 (0.1%) in PBS for 5 min. For anti-ERα Sp-1 (1∶1000) and lysotracker (75 nM) co-staining (Fig. 2F and Fig. S1B) cells were permeabilized with Triton-X 100 (0.1%) in PBS for 1 min. For anti-ERα HC-20 (1∶30) and lysotracker (75 nM) co-staining (Fig. 2H) cells were permeabilized with Triton-X 100 (0.1%) in PBS for 5 min. For anti-ERα Sp-1 (1∶1000) and EEA1 N-19 (1∶500) co-staining (Fig. 4A) cells were permeabilized with Triton-X 100 (0.1%) and saponin 0.01% in PBS for 1 min. For anti-ERα HC-20 (1∶30) and EEA1 H-300 (1∶1000) co-staining (Fig. 4B) cells were permeabilized with Triton-X 100 (0.1%) in PBS for 5 min. After the permeabilization process, cells were incubated with bovine serum albumin (BSA) (2%) for 30 minutes and than stained with the appropriate antibodies (see above) for 1 hour at room temperature. After that cells were rinsed three times in PBS for 5 minutes and incubated 30 min with Alexa Fluor 546, Alexa Fluor 488 donkey anti-rabbit secondary antibodies (1∶400), Alexa Fluor 546, Alexa Fluor 488 donkey anti-mouse secondary antibodies or Alexa Fluor 488® donkey anti-goat secondary antibodies (1∶400) (Invitrogen, Carlsbad, CA, USA) (1∶400) according to the specific co-staining protocol. Following extensive washes coverslips were mounted and confocal analisys was performed using LCS (Leica Microsystems, Heidelberg, Germany). Lysotracker red DND-99 (Invitrogen, Carlsbad, CA, USA) was incubated before fixation to live cells for 2 hrs at 37°C in the presence or in the absence of E2 stimulation.

### Statistical Analysis

A statistical analysis was performed using the ANOVA test with the InStat version 3 software system (GraphPad Software Inc., San Diego, CA). Densitometric analyses were performed using the freeware software Image J by quantifying the band intensity of the protein of interest respect to the relative loading control band (*i.e.,* vinculin or tubulin) intensity. In all analyses, *p* values <0.01 were considered significant, but for densitometric analyses where *p* was <0.05. Data are means of at least three independent experiments +/− SD.

## Supporting Information

Figure S1 **The involvement of lysosomes in E2-induced cell proliferation.** T47D-1 cells were co-stained with anti-ERα Sp-1 antibody together with either LAMP-2 antibody (A) or lysotracker (B) both in the presence and in the absence of E2 (10 nM–2 hrs). Figures show one unique confocal plane. All co-staining procedures were described in details in the Material and Methods section. (C) Time course analysis of T47D-1 cells treated with E2 (10 nM) at the indicated time points both in the presence and in the absence of chloroquine (Clo–10 µM). Loading control was done by evaluating vinculin expression in the same filter. Figure shows representative blots of three independent experiments. (D) Western blot analysis of cyclin D1 (Cyc D1) and Bcl-2 expression levels in T47D-1 cells treated with E2 (10 nM–24 hours) both in the presence and in the absence of chloroquine (Clo–10 µM). Loading control was done by evaluating tubulin expression in the same filter. Figure shows representative blots of three independent experiments.
(TIF)

Figure S2 **H2_NES ERα characterization.** (A) Schematic of the point mutations introduced in the hinge region of the ERα [21]. (B) pc DNA flag ERα and ERα (Nessi)-transfected HeLa cells were stained with anti-flag antibody. Figures show one unique confocal plane. All staining procedures were described in details in the Material and Methods section.
(TIF)
