# Supplementary figures and images for: Lysosomal Function Is Involved in 17β-Estradiol-Induced Estrogen Receptor α Degradation and Cell Proliferation
Source: PLoS One. 2014 Apr 15;9(4):e94880. doi: 10.1371/journal.pone.0094880 (PMC3988130; doi:10.1371/journal.pone.0094880)

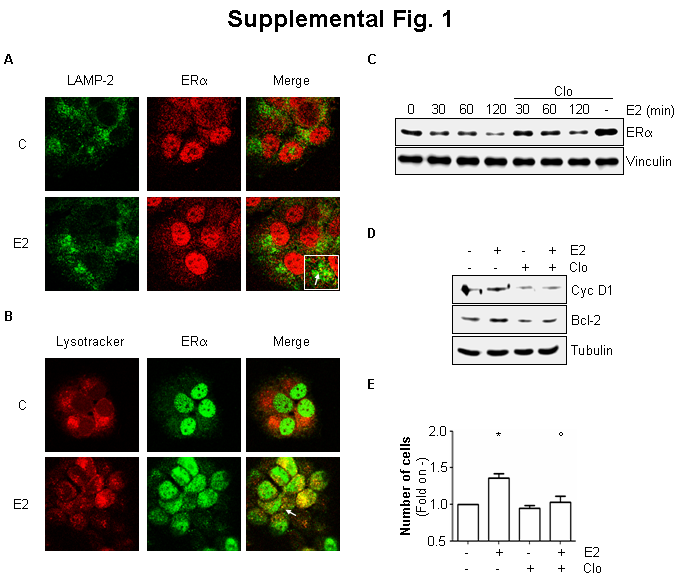

Supplement: Figure S1 — The involvement of lysosomes in E2-induced cell proliferation. T47D-1 cells were co-stained with anti-ERα Sp-1 antibody together with either LAMP-2 antibody (A) or lysotracker (B) both in the presence and in the absence of E2 (10 nM–2 hrs). Figures show one unique confocal plane. All co-staining procedures were described in details in the Material and Methods section. (C) Time course analysis of T47D-1 cells treated with E2 (10 nM) at the indicated time points both in the presence and in the absence of chloroquine (Clo–10 µM). Loading control was done by evaluating vinculin expression in the same filter. Figure shows representative blots of three independent experiments. (D) Western blot analysis of cyclin D1 (Cyc D1) and Bcl-2 expression levels in T47D-1 cells treated with E2 (10 nM–24 hours) both in the presence and in the absence of chloroquine (Clo–10 µM). Loading control was done by evaluating tubulin expression in the same filter. Figure shows representative blots of three independent experiments. (TIF) [file pone.0094880.s001.tif]

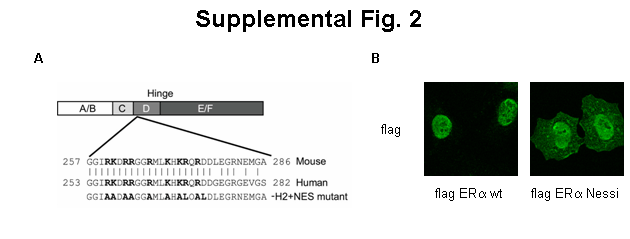

Supplement: Figure S2 — H2_NES ERα characterization. (A) Schematic of the point mutations introduced in the hinge region of the ERα [21]. (B) pc DNA flag ERα and ERα (Nessi)-transfected HeLa cells were stained with anti-flag antibody. Figures show one unique confocal plane. All staining procedures were described in details in the Material and Methods section. (TIF) [file pone.0094880.s002.tif]
